# A 4-month dataset of interactive SSH honeypot logs and command payloads

**DOI:** 10.1016/j.dib.2026.112860

**Published:** 2026-05-16

**Authors:** Viktor Boiko, Oleksandr Niiakyi

**Affiliations:** Faculty of Cybersecurity and Information Technologies, National University "Odesa Law Academy", Odesa, Ukraine

**Keywords:** SSH Honeypot, Cybersecurity, Network Security, Attack Dataset, Botnet Behavior, Command Payloads, Intrusion Detection

## Abstract

This article describes a longitudinal dataset of high-interaction SSH honeypot logs collected over a four-month period from July to November 2025. The data was captured using a custom modular multi-threaded honeypot developed in Python 3.10, utilizing the Paramiko library to simulate an interactive SSHv2 environment. Unlike low-interaction systems, this dataset records the full spectrum of adversary activity, including initial connection attempts, authentication brute-forcing, and post-authentication command execution. The resulting dataset contains 145,425 security events, structured to support session reconstruction via unique identifiers [1]. The telemetry includes source IPv4 addresses, credential pairs, and granular command-line payloads, featuring rare instances of file-less exploitation via bash sockets. This data provides a ground-truth source for training machine learning models for anomaly detection, enhancing SIEM rule-sets, and conducting empirical research into botnet orchestration and manual intrusion tactics in cloud infrastructures.

Specifications Table

**Table:** 

Subject	Computer Sciences
Specific subject area	Intrusion Detection, SSH Botnet Behavior Analysis
Type of data	Database (SQLite .db), Table (CSV), Figure, Graph.
Data collection	Custom-built modular multi-threaded SSH honeypot (Python 3.10)
Data source location	Odesa, Ukraine (Research) / Amsterdam, Netherlands (Deployment VPS)
Data accessibility	Repository name: Zenodo Data identification number: 10.5281/zenodo.19629700 Direct URL to data: https://doi.org/10.5281/zenodo.19629700 [1] Source code identification number: 10.5281/zenodo.19763025 Direct URL to source code: https://doi.org/10.5281/zenodo.19763025 [3]
Related research article	None

## 1 Value of the Data

• The 4-month longitudinal scope (July–November 2025) enables the identification of temporal anomalies, such as the observed daily surge in connection attempts at 13:00 UTC. This serves as a benchmark for studying global botnet orchestration and the impact of cron-scheduled reconnaissance on network infrastructure.
• The dataset provides high-fidelity telemetry of post-authentication behavior, including raw command-line payloads. This allows researchers to differentiate between automated "background radiation" and high-intent, targeted intrusion attempts.
• The observed class imbalance (94.6% INFO vs. 5.4% WARNING) reflects the real-world signal-to-noise ratio in modern cloud-based SSH environments. This distribution provides a realistic benchmark for developing anomaly detection systems that must operate under conditions of heavy background reconnaissance.
• Captured payloads include rare file-less delivery signatures, specifically the use of /dev/tcp bash sockets. These records provide essential ground-truth data for developing and validating signature-based and heuristic detection rules in Intrusion Detection Systems (IDS) and SIEM platforms.
• The inclusion of unique session identifiers (UUIDs) across all 145,425 records facilitates complete "kill chain" reconstruction, enabling researchers to trace the full lifecycle of an attack from initial brute-force to final payload deployment.
• The data is pre-cleaned to remove administrative noise and formatted in sqlite3 and csv, making it directly compatible with machine learning pipelines for training anomaly detection models on session-based temporal and behavioral features.

## 2 Background

The primary motivation for collecting this dataset was to provide an updated, longitudinal telemetry source from a Linux-based cloud environment. This project addresses the requirement for high-interaction data that captures modern botnet tactics, such as file-less payload delivery via /dev/tcp bash sockets and coordinated command execution, which remains a critical challenge in the evolution of cyber deception systems [2]. A custom modular honeypot was developed using Python 3.10 and the Paramiko library. The implementation utilizes a multi-threaded architecture to handle concurrent SSHv2 interactions and capture structured telemetry. The dataset serves as a ground-truth source for training machine learning models for anomaly detection and enhancing SIEM systems. Furthermore, the longitudinal nature of the data allows for the empirical study of botnet behavior, including temporal activity patterns, credential recycling, and the orchestration of automated scanning campaigns across cloud infrastructures.

## 3 Data Description

The dataset is publicly hosted on Zenodo[1] and consists of three primary components that provide different levels of granularity for the captured telemetry. The data includes a total of 145,425 security events collected over a four-month period.

• honey_db.tar.gz: A compressed archive containing original raw database honey.db (SQLite) with the collected logs.
• honey_csv.tar.gz: A compressed archive containing two structured representations of the data for general accessibility:
  ° ssh_attacks_full.csv: A complete, flattened export of the entire database. This file contains all 145,425 records, including transport-layer handshakes and connection metadata.
  ° ssh_attacks_warning.csv: A filtered dataset containing 4825 high-value security events, excluding background connection noise and focusing on authentication attempts and command execution payloads.
• geospatial_attack_distribution.csv: Supplemental metadata featuring geolocation and organizational data (via ipinfo.io).

The data is organized within a single primary table named honey. The technical specifications and definitions for each data field are detailed in Table 1 below.

**Table 1:** Description of the dataset fields.

Field	Description
Id	Unique primary key for each log entry.
session_id	UUID used to group events into a single attacker session.
timestamp	Date and time of the event in ISO 8601 format (UTC).
event_type	Interaction category: connect, login, disconnect, or exec_command.
ip	Source IPv4 address.
port	Source TCP port.
user	Username attempted during authentication.
password	Password attempted during authentication.
message	Raw metadata or system messages.
command	Shell command or payload string captured during execution.

**Fig. 1:**
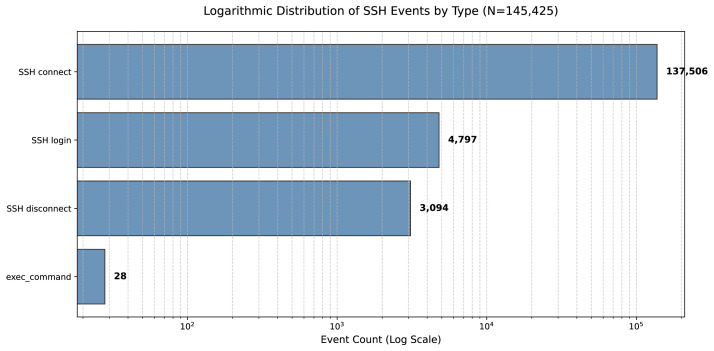
Logarithmic Distribution of SSH Events by Type.

**Fig. 2:**
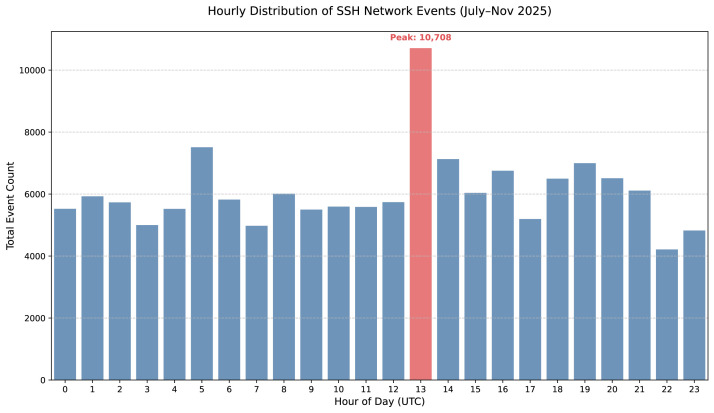
Hourly Distribution of SSH Network Events.

**Fig. 3:**
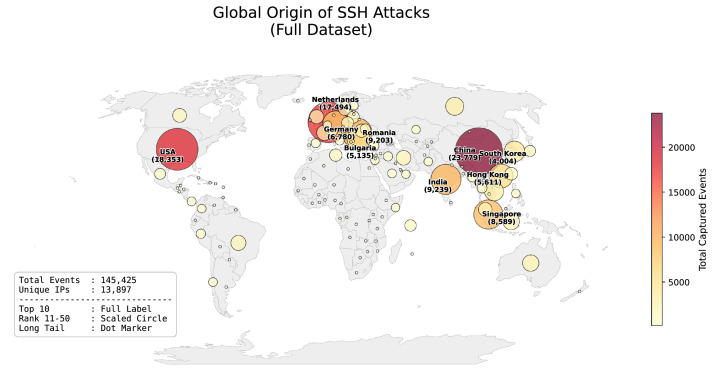
Global distribution of captured SSH attack events by origin country.

The event distribution follows a heavy-tailed pattern, where automated transport-layer noise (n = 145,425) significantly outweighs high-value application-layer data, as illustrated in Fig. 1. This dataset includes 4797 authentication events and 28 interactive command executions. Temporal analysis indicates a distinct activity surge at 13:00 UTC (Fig. 2). Fig. 3 illustrates the geographic distribution of 145,425 SSH attack events using a bubble map. Each marker corresponds to a country of origin identified via IP geolocation. To ensure clarity despite high data density, the visualization categorizes countries into three tiers: the top ten sources (e.g., China, USA, Netherlands) are marked with circles scaled to volume and labeled with both country names and event counts. Countries ranked 11th to 50th are represented by scaled circles without labels, while the remaining countries are indicated by uniform dots, representing the global "long tail" of the distribution.

Table 2 summarizes the top 10 regions by event volume, highlighting significant discrepancies between total activity and the number of unique IP addresses. The data for Bulgaria (BG) indicates a high concentration of activity originating from a limited number of sources. While 27 unique IPs were recorded, two specific nodes (IP: 87.120.191.13 and IP: 78.128.112.74) accounted for 97.91% of the 5135 regional events. Further granular analysis of attack signatures, including the most active source identifiers and the specific credential profiles used during brute-force attempts, is provided in Table 3. Representative payloads illustrating various attack stages are summarized in Table 4.

**Table 2:** Top 10 countries by event volume and unique IP intensity.

Country	ISO Code	Total Events	Unique IPs	Events per IP (Avg)
China	CN	23,779	2955	8.0
USA	US	18,353	2519	7.3
Netherlands	NL	17,494	484	36.1
India	IN	9239	536	17.2
Romania	RO	9203	64	143.8
Singapore	SG	8589	815	10.5
Germany	DE	6780	498	13.6
Hong Kong	HK	5611	735	7.6
Bulgaria	BG	5135	27	190.2
South Korea	KR	4004	425	9.4

**Table 3:** Statistical summary of the most active attack sources and credential profiles.

Category	Top Identifier / Profile	Count / Event Type	Country / Context
**IP Address**	87.120.191.13	4462 events	Bulgaria
**IP Address**	185.156.73.233	2096 events	Netherlands
**IP Address**	196.251.85.101	1940 events	Netherlands
**IP Address**	148.72.158.192	1870 events	USA
**IP Address**	143.110.248.222	1635 events	India
**Credentials**	345gs5662d34: 345gs5662d34	560 attempts	Generic Bruteforce
**Credentials**	root: 3245gs5662d34	383 attempts	Default Root Probing
**Credentials**	root: root	33 attempts	Common Default
**Credentials**	(null): (null)	28 attempts	Empty Auth / Logic Probe
**Credentials**	array: admin	20 attempts	Service Specific

**Table 4:** Representative examples of captured command payloads.

Category	Payload Example	Significance
Exploitation	nohup bash -c "exec 6<>/dev/tcp/…" &	Advanced file-less payload delivery
Persistence	dd bs=1 count=1911,588 > /tmp/djJJJF5kbk	Binary download and persistence prep
Reconnaissance	cd /tmp / cd /var	Manual navigation and system exploration
Human Interaction	учше (typo for 'ls'/'exit')	Human-driven operational errors

## 4 Experimental Design, Materials and Methods

The collection system was built using a custom multi-threaded framework, which is available on both Zenodo [3] and GitHub [4]. The honeypot is implemented as a multi-threaded Python 3.10 application using the Paramiko library [5]. The core logic handles SSHv2 transport and authentication layers by subclassing paramiko.ServerInterface. Incoming connections are encapsulated in individual threads, where each session is assigned a unique UUID. Command execution requests are intercepted via the check_channel_exec_request method, allowing for the capture of raw payloads without executing them on the host system. Data is persisted to a SQLite 3 database in real-time using a synchronous write-ahead logging approach.

To facilitate efficient data analysis and rapid telemetry pruning, the system implements a standardized two-tier logging hierarchy reflected in the level field:

• Level INFO: Assigned to transport-layer events, such as initial TCP handshakes and session terminations. These records account for 94.6% of the dataset.
• Level WARNING: Reserved for active application-layer interactions, specifically authentication attempts and shell command execution. This classification allows researchers to immediately isolate the 5.4% of high-value records.

The experimental infrastructure was deployed on an isolated Virtual Private Server (VPS) hosted in a Tier-3 datacenter in Amsterdam, Netherlands (AMS3 region). The node was provisioned through DigitalOcean (AS14061). The administrative SSH service was reconfigured to port 1022 to ensure that all traffic directed to port 22 was handled by the honeypot engine. To prevent weaponization, a strict "deny-all" egress filtering policy was applied using UFW, allowing only essential protocol traffic: DNS (UDP/53) and HTTP/HTTPS (TCP/80, 443).

To ensure maximum data purity, a comprehensive audit was conducted in April 2026. During this process, 74 internal administrative sessions (originating from localhost or 127.0.0.1) were identified and purged. This ensures that the final release (Version 2.1) exclusively represents external threat intelligence. The refined dataset has been published to the Zenodo repository under an open-access license [1].

The provided CSV part of dataset is encoded in standard UTF-8. To ensure data integrity during the transition from the SQLite database to flat-file formats, we utilized the standard SQLite CLI export mechanism (sqlite3 -csv), which correctly handles field escaping for payloads containing special characters or delimiters. While the CSV exports are sanitized for general use, we recommend that researchers requiring absolute byte-level integrity for the analysis of binary-obfuscated commands utilize the provided SQLite database (honey.db) directly, as it preserves the original raw payloads without the formatting constraints of text-based files.

## Limitations

The data collection concluded on November 14, 2025. The final date represents a partial observation window as the honeypot was decommissioned during the early hours. While the final observation window was partial due to the scheduled decommissioning of the honeypot in the early morning hours, this does not affect the statistical validity of the hourly activity patterns presented in Fig. 2. The temporal analysis reflects the aggregated behavioral characteristics of the captured traffic over the entire four-month observation period.

The data was collected from a single VPS node in Amsterdam (Netherlands). While the traffic is global, the volume and nature of the attacks may be influenced by the IP reputation and regional proximity of the host within the cloud provider's network.

The dataset preserves all captured telemetry in its original state within the **message** and **command** fields to ensure forensic integrity. The authentication data, including credential pairs, is recorded exactly as received by the honeypot engine without normalization or pre-filtering. Consequently, researchers must account for potential encoding artifacts, non-standard character sets, or empty strings that may occur when automated botnets submit binary or obfuscated payloads via the SSH protocol.

## Ethics Statement

The authors have read and follow the ethical requirements for publication in Data in Brief and confirming that the current work does not involve human subjects, animal experiments, or any data collected from social media platforms.

## CRediT Author Statement

Viktor Boiko: Conceptualization, Methodology, Data curation, Visualization, Writing, Original draft preparation, Editing. Oleksandr Niiakyi: Software, Validation, Investigation.

## Data Availability

ZenodoA 4-Month Dataset of SSH Botnet Interactions and Command Payloads (Original data)
